# Tea consumption and the risk of abdominal aortic aneurysm

**DOI:** 10.1093/bjs/znab468

**Published:** 2022-03-03

**Authors:** Joanna Kaluza, Otto Stackelberg, Holly R. Harris, Martin Björck, Alicja Wolk

**Affiliations:** 1 Unit of Cardiovascular and Nutritional Epidemiology, Institute of Environmental Medicine, Karolinska Institutet, Stockholm, Sweden; 2 Department of Human Nutrition, Warsaw University of Life Sciences–SGGW, Warsaw, Poland; 3 Department of Surgical Sciences, Unit of Medical Epidemiology, Uppsala University, Uppsala, Sweden; 4 Section of Vascular Surgery, Department of Clinical Science and Education, Karolinska Institutet at Södersjukhuset, Stockholm, Sweden; 5 Program in Epidemiology, Division of Public Health Sciences, Fred Hutchinson Cancer Research Center, Seattle, Washington, USA; 6 Department of Epidemiology, University of Washington, Seattle, Washington, USA; 7 Department of Surgical Sciences, Section of Vascular Surgery, Uppsala University, Uppsala, Sweden

## Abstract

**Background:**

Tea has the potential to lower the risk of abdominal aortic aneurysm (AAA) owing to its high antioxidant capacity. AAA risk factors including smoking, hypertension, and hypercholesterolaemia, may modify this association.

**Methods:**

The study population included 45 047 men in the Cohort of Swedish Men (COSM) and 36 611 women in the Swedish Mammography Cohort (SMC), aged 45–83 years at baseline. The COSM was established in 1997 with all men who lived in two central Swedish counties (Västmanland and Örebro), and the SMC was established in 1987–1990 with women residing in Västmanland county. Tea consumption was assessed by means of food frequency questionnaires in 1997 and 2009.

**Results:**

During 17.5 years of follow-up, 1781 AAA cases (1496 men, 285 women; 1497 non-ruptured, 284 ruptured) were ascertained via Swedish registers. Tea consumption was inversely associated with total AAA incidence in men and women. Women had a 23 (95 per cent c.i. 8 to 36) per cent lower risk of AAA per each cup per day increment, whereas men had a 9 (0 to 17) per cent lower risk (*P*_interaction_ = 0.029). Tea consumption was associated with a lower risk of both non-ruptured (hazard ratio (HR) 0.93, 95 per cent c.i. 0.85 to 1.01) and ruptured (HR 0.84, 0.71 to 0.98) AAA. Smoking status modified the association (*P*_interaction_ < 0.001), whereby tea consumption was associated with lower risk of AAA in ex-smokers (per cup per day, HR 0.89, 0.80 to 0.98) and in never smokers (HR 0.88, 0.77 to 1.00), but not in current smokers (HR 0.95, 0.84 to 1.06). Tea consumption was associated with a lower risk in participants with (HR 0.88, 0.80 to 0.98) and without (HR 0.93, 0.88 to 1.00) hypertension, and in those with (HR 0.82, 0.67 to 1.01) and without (HR 0.92, 0.86 to 0.98) hypercholesterolaemia.

**Conclusion:**

Tea consumption was associated with a reduced risk of AAA. The association was more pronounced for ruptured than non-ruptured AAA, and in patients with hypertension and hypercholesterolaemia than those without. The association was also observed in ex-smokers and never smokers, but not in current smokers.

## Teaser

The results of the study suggest that tea consumption may be associated with a reduced risk of abdominal aortic aneurysm (ruptured and non-ruptured) in men and women, in participants with and without hypertension and hypercholesterolaemia, and in never and ex-smokers but not in current smokers. Tea consumption has the potential for the prevention of abdominal aortic aneurysm and, in addition to quitting smoking, a healthy diet including tea could help reduce this risk.

## Introduction

Oxidative stress and inflammation have a dominant role in the aetiology of abdominal aortic aneurysm (AAA)^1^. Among known risk factors, smoking^2–5^ and hypertension^2,4^ are both related to oxidative stress^6,7^, whereas a role for hypercholesterolaemia remains unclear^8,9^. Results of a recently published meta-analysis^3^ indicate that current and ex-smokers have a 4.9- and 2.1-fold higher risk of AAA respectively compared with never smokers, whereas hypertension increases the risk by 66 per cent^10^.

Human studies have shown that tea is associated with a lower risk of some oxidative stress-related diseases including cardiovascular disease (CVD), such as coronary artery disease^11^ and myocardial infarction^12,13^, and BP^14^. However, the beneficial effect of tea consumption on blood cholesterol concentrations has not been confirmed^15,16^. The protective role of tea is attributed to its antioxidative properties related to the high content of flavonoids^17^, especially catechins, oligomeric flavonoids, and flavonols^18,19^. Although the health benefits of tea consumption in relation to CVD have been well documented^20,21^, and oxidative stress also underlies the aetiology of AAA, no previous studies have examined the impact of tea consumption on risk of AAA development.

The aim of this study was to prospectively examine the association between tea consumption and risk of total, non-ruptured, and ruptured AAA among men and women. The question of whether oxidative stress-related AAA risk factors such as smoking status, hypertension, and hypercholesterolaemia modified the observed association was also addressed.

## Methods

### Study population

The study population included the Cohort of Swedish Men (COSM) and the Swedish Mammography Cohort (SMC). The COSM was established in the fall of 1997 when all men born in 1918–1952 who lived in two counties in central Sweden (Västmanland and Örebro) were invited to join. The SMC was established in 1987–1990 when all women born in 1914–1949, who were residing in Västmanland and Uppsala counties, were invited to participate in a mammography screening programme.

**Fig. 1:**
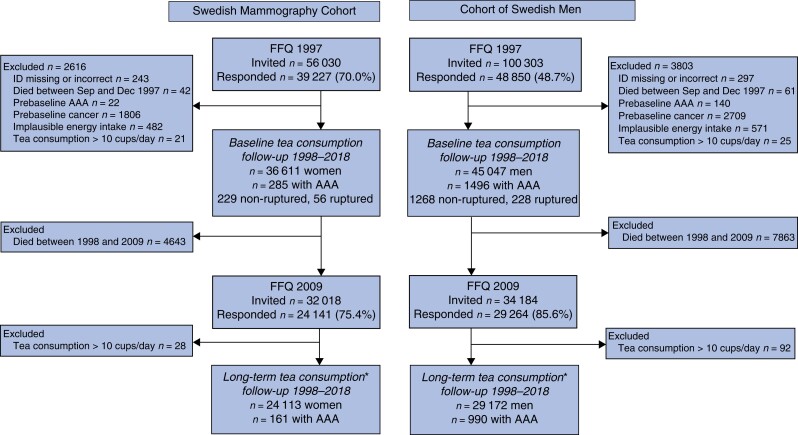
Flow chart of Swedish Mammography Cohort and Cohort of Swedish Men

In late 1997, 48 850 men (48.7 per cent response rate) and 39 227 women (70.0 per cent response rate) completed a 96-item food frequency questionnaire (FFQ) and answered questions about other lifestyle factors (*Fig. 1*). In 2009, an expanded questionnaire containing 132 food items was sent to men and women from the COSM and SMC, of whom 85.6 and 75.4 per cent respectively responded. All of the questionnaires used in this study are available online at https://www.simpler4health.se/researchers/questionnaires/. The study was approved by the Regional Ethical Review Board, Stockholm, Sweden. Completion and return of the questionnaire were considered as informed consent to participate in the study.

### Exclusions and follow-up of cohorts

Of the 48 850 men and 39 227 women who completed the 1997 questionnaire, participants with an incorrect or missing personal identity number (297 men, 243 women), those who died before 1 January 1998 (the start of follow-up) (61 men, 42 women), and those with prevalent AAA (140 men, 22 women) or prevalent cancers other than non-melanoma skin cancer (2709 men, 1806 women) were excluded (*Fig. 1*). Moreover, participants with extreme energy intake (±3 standard deviations from the mean value of the log_e_-transformed estimates calculated separately by sex; 571 men, 482 women) and those with extremely high tea consumption (over 10 cups/day in 1997; 25 men, 21 women) were excluded. After these exclusions, 45 047 men and 36 611 women were included in the baseline analytical cohorts.

For analysis of long-term tea consumption, of 29 264 men and 24 141 women who completed the 2009 questionnaire, participants with extremely high tea consumption (over 10 cups/day in 2009; 92 men, 28 women) were excluded. The long-term analytical cohorts included 29 172 men and 24 113 women.

### Assessment of tea consumption

Data on food consumption were collected using the validated 96-item FFQ in 1997^22^ and the expanded 132-item FFQ in 2009. The 1997 FFQ included one question about total tea consumption, whereas the 2009 FFQ included three questions, on consumption of black, green, and herbal/red tea. Using open-ended questions, participants were asked to indicate how often, on average (mean), they drank tea on a daily or weekly basis (1997 FFQ) or specific tea (2009 FFQ) during the past year. To take into account potential changes in frequency of tea consumption, results for long-term tea consumption are also presented. Long-term tea consumption was estimated for each participant by calculating the mean total tea consumption from the questionnaires in 1997 and 2009. In a validation study, comparing the FFQ with four 1-week weighted diet records of 129 women from the SMC, the Spearman correlation coefficient for total tea consumption was 0.81 (A. Wolk, unpublished data).

### Assessment of co-variables

Information on level of education, body height and weight, time per day spent walking and/or cycling, history of CVD, family history of myocardial infarction before 60 years old, and aspirin use were collected in the 1997 questionnaire. Respondents were also asked about smoking history, age when they started smoking, mean number of cigarettes smoked daily for specific age categories (aged 15–20, 21–30, 31–40, 41–50, and 51–60 years, and current age), and, if applicable, the age they quit smoking. Pack-years were calculated by multiplying the number of years of smoking by the reported daily number of cigarettes smoked within respective age categories. Data on hypertension, hypercholesterolaemia, and diabetes diagnoses were collected through self-report in the questionnaire in 1997 and by linkage with health registers. Hypertension and hypercholesterolaemia cases were ascertained via linkage with the National Patient Register (ICD-10 codes: I109 for hypertension; E78.0, E78.2, and E78.4 for hypercholesterolaemia), and diabetes cases via linkage with the Swedish National Diabetes Register and the National Patient Register (ICD-10 codes: E10–E14). Energy intake was calculated by multiplying the frequency of consumption by age- and sex-specific portion sizes and by the energy content of each food item (obtained from the Swedish Food Administration Database)^23^. A modified Mediterranean diet (mMED) score was calculated to assess total quality of diet, as described previously^24^. The mMED score ranged from 0 to 8 points; a larger number of points corresponded to greater adherence to the Mediterranean diet. Information on statin use was obtained from the National Swedish Registry of Prescriptions and was available from 2006. Therefore, in the analysis adjusted for statin use, follow-up was limited to 2006–2018 and the cohorts were limited to 36 636 men and 31 967 women with 1022 and 170 AAAs respectively.

### Ascertainment of abdominal aortic aneurysm

Incident cases of AAA, AAA repair, and death from AAA were identified by linkage of the cohorts with the Swedish Inpatient Register and the Swedish National Cause of Death Register. To identify non-ruptured and ruptured aneurysm in the abdominal aorta, ICD-10 codes I71.4 and I71.3 respectively were used. AAA repair was identified from the Swedish Inpatient Register by use of the Nordic Medico-Statistical Committee Classification of Surgical Procedures, and the Swedish National Registry for Vascular Surgery (Swedvasc) by use of the integrated AAA module in that registry. The Swedish Inpatient Register has not been validated specifically for AAA; however, it has a high validity in general^25^ and nearly complete data on hospitalizations for the Swedish population since 1987^26^. Surgical procedures have been reported as incorrect in 2 per cent, and missing in 5.3 per cent, of the records^25^. In a recent independent international validation of the Swedvasc registry, the external validity was 98.8 per cent for AAA repair^27^.

Analyses of a subcohort of 10 536 men from the COSM who were screened for AAA during follow-up as a part of the screening programme of men aged 65–75 years (initiated in 2006) were undertaken. Examinations were performed either by an ultrasound technician or a registered nurse with specific ultrasound proficiency. The outer-to-outer method was used in Västmanland and Örebro counties until 2011, after which the leading edge-to-leading edge method was adopted throughout Sweden. Differences between the two methods were accounted for by subtracting 2 mm from all measurements measured by the outer-to-outer method before analysis. In this screened subcohort, 168 men (1.6 per cent) were classified as having an AAA, defined as an infrarenal aortic diameter of 30 mm or more^28,29^.

### Statistical analysis

Cox proportional hazards regression models were used to estimate hazard ratios (HRs) with 95 per cent confidence intervals for all AAAs, and separately for non-ruptured and ruptured aneurysms. Participants were followed from 1 January 1998 (baseline) to the date of diagnosis of AAA or AAA repair, death, or the end of follow-up (31 December 2018), whichever occurred first. Tea consumption was categorized as: 0, 0.1–0.9, 1.0–1.9, or at least 2.0 cups/day. Multivariable models were adjusted for age at study baseline (years, continuous), sex, education (primary, high school, or university), smoking status (never; ex-smoker: less than 20, 20–39, or at least 40 pack-years; or current: less than 20, 20–39, or at least 40 pack-years), occupation (full-time, part-time, retired, disability pension, unemployed, or housewife), BMI (less than 20.0, 20.0–24.9, 25.0–29.9, or at least 30 kg/m^2^), walking/cycling (less than 20, 20–40, 40–60, or over 60 min/day), aspirin use (yes, no), history of hypertension (yes, no), diabetes (yes, no), hypercholesterolaemia (yes, no), CVD (yes, no), family history of myocardial infarction (yes, no), coffee consumption (no more than 1.0, 1.1–3.0, 3.1–5.0, or over 5.0 cups/day), sugar consumption (0, 0.1–1.0, 1.1–3.0, 3.1–5.0, or at least 5.1 teaspoons/day), modified Mediterranean diet score (points, continuous), and energy intake (kcal/day, continuous). In separate analyses of black, green, and herbal/red tea, the specific types of tea were mutually adjusted for. Missing data were included in the models as separate categories and concerned data on educational level (0.5 per cent), occupation (0.5 per cent), smoking status (1.6 per cent), BMI (3.6 per cent), walking/cycling (8.7 per cent), and aspirin use (10.7 per cent). Adjustment was also made for statin use (0, 1, 2–3, 4–5, 6–10, or over 10 years) in the multivariable models. This information was available only from 2006 to 2018.

The assumption of proportional hazards was tested by regressing scaled Schoenfeld residuals against survival time, and no evidence of departure from the assumption was found. Interactions on the multiplicative scale between categories of tea consumption by sex and smoking status were tested using a likelihood ratio test. Linear trends were assessed by including cups of tea consumed per day in the models as a continuous variable. Non-linear trends were investigated using restricted cubic splines with three knots (at the 10th, 50th, and 90th percentiles calculated among tea drinkers)^30^.

The association of tea consumption in relation to infrarenal aortic diameter (less than 30 mm, or 30 mm or more) was assessed using a multivariable logistic regression model in the screening subcohort of the COSM. The model was adjusted for the same co-variables as the Cox proportional hazards models, except age; age at screening instead of age at the study baseline was included.

All statistical analyses were conducted using Stata^®^ software, version 14 (StataCorp, College Station, TX, USA). All reported *P* values are two-sided and *P* ≤0.050 was considered statistically significant.

## Results

During a mean(s.d.) of 17.5(5.5) years of follow-up from 1 January 1998 to 31 December 2018 (1 427 054 person-years), 1781 incident AAA cases were identified, including 1497 (84.0 per cent) non-ruptured and 284 (16.0 per cent) ruptured AAAs (*Fig. 1*). Most of the ascertained AAAs were in men (84.0 per cent) and smokers (current 45.2 per cent, ex-smokers 36.4 per cent).

*Long-term tea consumption calculated as mean tea consumption based on data from 1997 and 2009 food frequency questionnaires (FFQ). AAA, abdominal aortic aneurysm.

Approximately half of men and women (51 per cent) did not report tea consumption, whereas 13 per cent consumed two or more cups per day (*Table 1*). The mean(s.d.) frequency of tea consumption among drinkers was 1.3(1.3) cups/day in men and 1.3(1.2) cups/day in women, which corresponded to consumption of 351(347) and 294(287) ml/day respectively. Compared with non-drinkers (0 cups/day), participants in the highest category of tea consumption (at least 2.0 cups/day) were more likely to have a university education, less likely to be current smokers, less likely to have hypertension, diabetes, hypercholesterolaemia or CVD, and had a lower mean BMI (*Table 1*).

**Table 1:** Age-standardized baseline characteristics of 45 047 men from the Cohort of Swedish Men and 36 611 women from the Swedish Mammography Cohort by categories of tea consumption, 1997 (baseline)

	Tea consumption (range (median) servings/day)			
	0	0.1–0.9 (0.3)	1.0–1.9 (1.0)	≥2.0 (2.0)
**No. of people**	41 542	15 313	14 157	10 646
**Total no. with AAA**	1106	250	268	157
**Total no. with ruptured AAA**	176	41	51	16
**Men**	24 173 (58.3)	7843 (51.0)	7510 (53.1)	5521 (51.7)
Age (years)*	61.6 (9.6)	59.8 (9.1)	61.0 (9.6)	60.0 (9.4)
**University education**	5252 (13.1)	2629 (16.3)	2780 (19.6)	3426 (31.1)
**Occupation**				
Full-time	15 613 (39.7)	6786 (40.6)	5848 (41.2)	4773 (41.8)
Part-time	3386 (8.5)	1627 (9.9)	1342 (9.5)	993 (8.9)
Retired	17 023 (38.2)	5142 (38.6)	5445(38.4)	3670 (38.3)
Disability	2870 (7.1)	846 (5.2)	743 (5.3)	590 (5.4)
Unemployed	1742 (4.4)	598 (3.6)	492 (3.5)	388 (3.4)
Other	693 (1.6)	246 (1.7)	239 (1.7)	190 (1.8)
**Smoking status**				
Current smoker	11 594 (28.3)	3192 (20.4)	2 690 (19.0)	1868 (17.3)
Ex-smoker	13 057 (31.6)	4748 (30.7)	4531 (32.0)	3207 (30.0)
Never smoker	16 169 (38.4)	7164 (47.5)	6743 (47.6)	5413 (51.2)
BMI (kg/m^2^)*	25.7 (3.7)	25.4 (3.6)	25.2 (3.5)	24.9 (3.6)
**Walking/cycling (min/day)**				
< 20	13 488 (32.5)	4637 (30.2)	4377 (30.9)	3069 (28.7)
20–40	11 230 (27.1)	4698 (30.5)	4264 (30.1)	3297 (30.8)
40–60	6056 (14.5)	2508 (16.4)	2198 (15.6)	1726 (16.3)
> 60	6719 (16.0)	2367 (15.8)	2186 (15.5)	1719 (16.5)
**Hypertension**	10 308 (24.2)	3380 (23.0)	3337 (23.6)	2176 (21.1)
**Diabetes**	3629 (8.4)	976 (6.8)	1020 (7.2)	705 (6.9)
**Hypercholesterolaemia**	5657 (13.5)	1955 (12.9)	1808 (12.8)	1154 (11.0)
**Cardiovascular disease**‡	5083 (11.6)	1363 (9.9)	1556 (10.9)	967 (9.8)
Ischaemic heart disease	3399 (7.8)	924 (6.7)	1041 (7.3)	605 (6.2)
Heart failure	709 (1.6)	172 (1.3)	207 (1.4)	123 (1.3)
Stroke	1177 (2.7)	284 (2.1)	336 (2.4)	196 (2.0)
Atrial fibrillation	1095 (2.5)	311 (2.3)	383 (2.7)	268 (2.7)
**Family history of myocardial infraction**	5120 (12.4)	1863 (12.1)	1717 (12.1)	1318 (12.2)
**Aspirin use**	15 199 (36.6)	6188 (40.6)	5607 (39.6)	4294 (40.4)
Energy intake (kcal/day)*	2234 (865)	2203 (816)	2232 (802)	2376 (878)
Coffee consumption (cups/day)*	3.7 (2.1)	3.0 (1.8)	2.5 (1.7)	2.0 (1.8)
Sugar in tea/coffee (teaspoons/day)*	1.3 (2.5)	1.2 (2.1)	1.3 (2.2)	1.7 (2.6)
Modified Mediterranean diet score*	3.6 (1.6)	3.8 (1.6)	3.8 (1.6)	4.1 (1.6)
**Data collected using 2009 FFQ**†				
No. of people	25 672	10 737	9505	7371
Tea consumption (cups/day)				
Total tea	0 (0, 1.2)	0.3 (0, 2.1)	1.0 (0, 2.3)	1.1 (0, 4.6)
Black tea	0 (0, 1.1)	0.1 (0, 1.5)	0.5 (0, 2.3)	1.0 (0, 4.0)
Green tea	0 (0, 0.8)	0 (0, 1.1)	0 (0, 1.1)	0 (0, 2.0)

Values in parentheses are percentages unless indicated otherwise; values are *mean(s.d.) and †median (95 per cent c.i.). Missing data: educational level (0.5 per cent), occupation (0.5 per cent), smoking status (1.6 per cent), BMI (3.6 per cent), walking/cycling (8.7 per cent), and aspirin use (10.7 per cent). ‡Includes ischaemic heart disease (ICD-10: I20–I25), heart failure (I50, I110), stroke (I60, I61, I63, I64), and atrial fibrillation (I48).

Baseline tea consumption was inversely associated with the risk of AAA (*Table 2*). A significant interaction between tea consumption and sex in relation to risk of AAA was observed (*P*_interaction_ = 0.029). Each cup/day increment in tea consumption was associated with a 23 per cent lower risk in women and 9 per cent lower risk in men in the linear range of the association, that is for tea consumption of at least one cup/day. Although no statistically significant interaction was observed between tea consumption and age categories in relation to risk of AAA (*P*_interaction_ = 0.687), the association was stronger in the oldest group. The HR per cup daily increment of tea consumption was 0.94 (95 per cent c.i. 0.86 to 1.03; *P*_linear trend_ = 0.166) for participants aged less than 60 years, 0.93 (0.85 to 1.02; *P*_linear trend_ = 0.118) for those aged 60–69 years, and 0.87 (0.77 to 0.98; *P*_linear trend_ = 0.023) for those aged 70 years or more. Further adjustment for statin use in a subcohort with shorter follow-up (2006–2018, as data on statin use were lacking before 2006) did not changed the results markedly; the HR for total AAA per cup per day increment was 0.93 (0.86 to 0.98; *P*_linear trend_ = 0.012) for all participants, 0.93 (0.87 to 1.00; *P*_linear trend_ = 0.054) for men, and 0.82 (0.66 to 1.02; *P*_linear trend_ = 0.070) for women.

**Table 2:** Association between tea consumption and risk of abdominal aortic aneurysm (AAA) in 45 047 men (1496 with AAA) from the Cohort of Swedish Men and 36 611 women (285 with AAA) from the Swedish Mammography Cohort, follow-up 1998–2018

Tea consumption (range (median) cups/day)	Men and women					Men		Women	
	No. of AAAs	Person-years	SIR	Age-, sex- and smoking-adjusted HR	Multivariable HR*	No. of AAAs	Multivariable HR*	No. of AAAs	Multivariable HR*
**Baseline consumption (1997)**									
0	1106	706 516	157	1.00 (reference)	1.00 (reference)	922	1.00 (reference)	184	1.00 (reference)
0.1–0.9 (0.3)	250	277 798	90	0.78 (0.58, 0.90)	0.79 (0.69, 0.91)	206	0.82 (0.70, 0.95)	44	0.71 (0.51, 0.99)
1.0–1.9 (1.0)	268	248 671	108	0.88 (0.77, 1.01)	0.91 (0.79, 1.04)	231	0.97 (0.83, 1.12)	37	0.62 (0.43, 0.89)
≥2.0 (2.0)	157	191 661	82	0.73 (0.62, 0.86)	0.77 (0.65, 0.92)	137	0.83 (0.69, 1.01)	20	0.52 (0.32, 0.84)
*P* for non-linear trend				0.031	0.019		0.033		
Per cup‡				0.89 (0.82, 0.97)	0.90 (0.82, 0.98)		0.91 (0.83, 1.00)		
*P* for linear trend									0.004
Per cup									0.77 (0.64, 0.92)
**Long-term consumption (1997 and 2009)†**									
0	473	316 007	150	1.00 (reference)	1.00 (reference)	409	1.00 (reference)	64	1.00 (reference)
0.1–0.9 (0.3)	421	429 831	98	0.89 (0.78, 1.02)	0.93 (0.81, 1.07)	350	0.92 (0.79, 1.06)	71	1.03 (0.72, 1.45)
1.0–1.9 (1.0)	182	201 825	90	0.84 (0.71, 1.00)	0.90 (0.75, 1.07)	165	0.96 (0.80, 1.16)	17	0.56 (0.32, 0.98)
≥2.0 (2.0)	75	103 880	72	0.72 (0.57, 0.92)	0.78 (0.61, 1.01)	66	0.82 (0.62, 1.07)	9	0.68 (0.32, 1.41)
*P* for linear trend				< 0.001	0.017		0.093		0.053
Per cup				0.80 (0.74, 0.87)	0.91 (0.85, 0.98)		0.93 (0.86, 1.01)	161	0.79 (0.62, 1.00)

Values in parentheses are 95 per cent confidence intervals unless indicated otherwise. *Adjusted for age at study baseline (years, continuous), sex, education (primary, high school, or university), occupation (full-time, part-time, retired, disability pension, unemployed, or housewife), smoking status (never; ex-smoker: less 20, 20–39, or at least 40 pack-years; or current: less than 20, 20–39, or at least 40 pack-years), BMI (less than 20.0, 20.0–24.9, 25.0–29.9, or at least 30.0 kg/m^2^), walking/cycling (less than 20, 20–40, 40–60, or over 60 min/day), history of hypertension (yes, no), diabetes (yes, no), hypercholesterolaemia (yes, no), and cardiovascular diseases (yes, no), family history of myocardial infarction (yes, no), aspirin use (yes, no), coffee consumption (no more than 1.0, 1.1–3.0, 3.1–5.0, or over 5.0 cups/day), sugar consumption (0, 0.1–1.0, 1.1–3.0, 3.1–5.0, or at least 5.1 teaspoons/day), modified Mediterranean diet score (points, continuous), and intake of energy (kcal/day, continuous). Missing data on educational level (0.5 per cent), occupation (0.5 per cent), smoking status (1.6 per cent), BMI (3.6 per cent), walking/cycling (8.7 per cent), and aspirin use (10.7 per cent) were included in the models as separate categories. †Long-term consumption calculated as mean tea consumption from 1997 and 2009 in 53 285 participants (29 172 men and 24 113 women) with 1151 abdominal aortic aneurysms (AAAs) (990 men and 161 women). ‡Calculated for linear range of association, that is tea consumption of at least one cup/day. SIR, standardized incidence rate; HR, hazard ratio.

For long-term total tea consumption (mean intake at baseline 1997 and in 2009) similar associations were observed, but were not statistically significant. Each cup/day increment in consumption was associated with a 21 per cent lower risk in women and 7 per cent lower risk in men (*Table 2*).

The risk of AAA development in relation to consumption of specific types of tea was examined using data from the 2009 FFQ only. Owing to limited statistical power, because few participants drank green or herbal/red tea, women and men were combined in the analysis. The HR per each cup/day increase was 0.89 (0.79 to 1.01) for green tea, 0.87 (0.73 to 1.04) for herbal/red tea, and 0.98 (0.91 to 1.04) for black tea.

### Association by rupture status

An inverse association between tea consumption and non-ruptured AAA was observed in women (22 per cent lower risk per cup per day increment) and a similar tendency was observed in men (6 per cent risk reduction per cup per day for tea consumption of at least 1 cup/day) (*Table 3*).

**Table 3:** Association of baseline tea consumption with risk of abdominal aortic aneurysm (AAA) stratified by rupture status in 45 047 men (1496 with AAA) from the Cohort of Swedish Men and 36 611 women (285 with AAA) from the Swedish Mammography Cohort, follow-up 1998–2018

Baseline tea consumption (range (median) cups/day)	Men and women			Men		Women	
	No. of AAAs	Person-years	Multivariable HR*	No. of AAAs	Multivariable HR*	No. of AAAs	Multivariable HR*
**Non-ruptured**							
0	930	706 910	1.00 (reference)	780	1.00 (reference)	150	1.00 (reference)
0.1–0.9 (0.3)	209	278 183	0.78 (0.67, 0.91)	177	0.82 (0.70, 0.97)	32	0.61 (0.41, 0.90)
1.0–1.9 (1.0)	217	249 372	0.88 (0.76, 1.03)	187	0.94 (0.79, 1.11)	30	0.60 (0.40, 0.89)
≥2.0 (2.0)	141	192 589	0.83 (0.69, 1.00)	124	0.90 (0.74, 1.10)	17	0.51 (0.30, 0.87)
*P* for non-linear trend			0.021		0.044		
Per cup†			0.93 (0.85, 1.01)		0.94 (0.85, 1.03)		
*P* for linear trend							0.012
Per cup							0.78 (0.64, 0.95)
**Ruptured**							
0	176	706 910	1.00 (reference)	142	1.00 (reference)	34	1.00 (reference)
0.1–0.9 (0.3)	41	278 183	0.87 (0.61, 1.23)	29	0.78 (0.52, 1.18)	12	1.18 (0.60, 2.31)
1.0–1.9 (1.0)	51	249 372	1.02 (0.74, 1.41)	44	1.11 (0.78, 1.57)	7	0.72 (0.31, 1.66)
≥2.0 (2.0)	16	192 589	0.49 (0.29, 0.84)	13	0.50 (0.27, 0.90)	3	0.54 (0.16, 1.83)
*P* for linear trend			0.030		0.101		0.141
Per cup			0.84 (0.71, 0.98)		0.87 (0.73, 1.03)		0.72 (0.46, 1.11)

Values in parentheses are 95 per cent confidence intervals unless indicated otherwise. *Adjusted for age at study baseline (years, continuous), sex, education (primary, high school, or university), occupation (full-time, part-time, retired, disability pension, unemployed, or housewife), smoking status (never; ex-smoker: less than 20, 20–39, or at least 40 pack-years; or current: less than 20, 20–39, or at least 40 pack-years), BMI (less than 20.0, 20.0–24.9, 25.0–29.9, or at least 30 kg/m^2^), walking/cycling (less than 20, 20–40, 40–60, or over 60 min/day), history of hypertension (yes, no), diabetes (yes, no), hypercholesterolaemia (yes, no), and cardiovascular diseases (yes, no), family history of myocardial infarction (yes, no), aspirin use (yes, no), coffee consumption (no more than 1.0, 1.1–3.0, 3.1–5.0, or over 5.0 cups/day), sugar consumption (0, 0.1–1.0, 1.1–3.0, 3.1–5.0, or at least 5.1 teaspoons/day), modified Mediterranean diet score (points, continuous), and intake of energy (kcal/day, continuous). Missing data on educational level (0.5 per cent), occupation (0.5 per cent), smoking status (1.6 per cent), BMI (3.6 per cent), walking/cycling (8.7 per cent), and aspirin use (10.7 per cent) were included in the models as separate categories. †Calculated for linear range of association, that is tea consumption of at least one cup/day. AAA, abdominal aortic aneurysm; HR, hazard ratio.

#### Subcohort with measured infrarenal aortic diameter

In the analysis of the subcohort of 8528 men with infrarenal aortic diameter measurements available, no association between tea consumption and infrarenal aortic diameter was observed; the multivariable odds ratio per cup was 0.97 (95 per cent c.i. 0.72 to 1.30) for tea consumption of at least one cup/day in men with an infrarenal aortic diameter of 30 mm or more *versus* less than 30 mm.

For ruptured AAA, a statistically significant lower risk was observed with increasing tea consumption among all participants; each cup/day increment was associated with a 16 per cent lower risk (*Table 3*). The association for each cup/day appeared to be stronger in women (28 per cent lower risk) than in men (13 per cent), but these sex-specific results did not reach statistical significance owing to small numbers.

### Association by smoking status

An interaction was observed between tea consumption and smoking status in relation to AAA incidence (*P*_interaction_ < 0.001). There was a statistically significant inverse association between tea consumption and AAA incidence in ex-smokers and never smokers, but not in current smokers (*Table 4*); there was an 11 per cent lower risk in ex-smokers, and a 12 per cent lower risk in never smokers for each cup/day increment.

**Table 4:** Association between baseline tea consumption and risk of abdominal aortic aneurysm (AAA) by smoking status in 45 047 men (1496 with AAA) from the Cohort of Swedish Men and 36 611 women (285 with AAA) from the Swedish Mammography Cohort, follow-up 1998–2018

Baseline tea consumption (range (median) cups/day)	Current smoker			Ex-smoker			Never smoker		
	No. of AAAs	Person-years	Multivariable HR*	No. of AAAs	Person-years	Multivariable HR*	No. of AAAs	Person-years	Multivariable HR*
**0**	532	191 511	1.00 (reference)	409	224 807	1.00 (reference)	156	278 913	1.00 (reference)
**0.1–0.9 (0.3)**	100	56 694	0.75 (0.61, 0.94)	87	86 158	0.74 (0.58, 0.94)	61	131 627	0.98 (0.72, 1.33)
**1.0–1.9 (1.0)**	107	45 751	0.97 (0.78, 1.20)	100	79 723	0.83 (0.66, 1.04)	59	120 581	0.95 (0.70, 1.29)
**≥2.0 (2.0)**	66	32 524	0.88 (0.67, 1.16)	53	57 946	0.67 (0.49, 0.91)	35	99 237	0.76 (0.51, 1.12)
***P* for non-linear trend**			0.037						
**Per cup†**			0.95 (0.84, 1.06)						
***P* for linear trend**						0.015			0.049
**Per cup**						0.89 (0.80, 0.98)			0.88 (0.77, 1.00)

Values in parentheses are 95 per cent confidence intervals unless indicated otherwise. *Adjusted for age at study baseline (years, continuous), sex, education (primary, high school, or university), occupation (full-time, part-time, retired, disability pension, unemployed, or housewife), BMI (less than 20.0, 20.0–24.9, 25.0–29.9, or at least 30 kg/m^2^), walking/cycling (less than 20, 20–40, 40–60, or over 60 min/day), history of hypertension (yes, no), diabetes (yes, no), hypercholesterolaemia (yes, no), and cardiovascular diseases (yes, no), family history of myocardial infarction (yes, no), aspirin use (yes, no), coffee consumption (no more than 1.0, 1.1–3.0, 3.1–5.0, or over 5.0 cups/day), sugar consumption (0, 0.1–1.0, 1.1–3.0, 3.1–5.0, or at least 5.1 teaspoons/day), modified Mediterranean diet score (points, continuous), and intake of energy (kcal/day, continuous). Missing data on educational level (0.5 per cent), occupation (0.5 per cent), BMI (3.6 per cent), walking/cycling (8.7 per cent) and aspirin use (10.7 per cent) were included in the models as separate categories. †Calculated for linear range of association, that is tea consumption of at least one cup/day. AAA, abdominal aortic aneurysm; HR, hazard ratio.

### Associations by hypertension and hypercholesterolaemia

There was an inverse association between tea consumption and risk of AAA in participants with and without hypertension and hypercholesterolaemia (*Table 5*). Each cup per day increment in tea consumption was associated with a 7 (95 per cent c.i. 0 to 12) per cent lower risk in participants without hypertension and an 8 (2 to 14) per cent lower risk in those without hypercholesterolaemia. Among participants with hypertension, each cup per day increment in tea consumption was associated with a 12 (2 to 20) per cent lower risk, and there was a similar tendency among participants with hypercholesterolaemia, with a risk reduction of 18 (–1 to 33) per cent.

**Table 5:** Association between baseline tea consumption and risk of abdominal aortic aneurysm (AAA) by hypertension and hypercholesterolaemia in 45 047 men (1496 with AAA) from the Cohort of Swedish Men and 36 611 women (285 with AAA) from the Swedish Mammography Cohort, follow-up 1998–2018

Baseline tea consumption (range (median) cups/day)	Disease absent			Disease present		
	No. with AAA	Person-years	Multivariable HR*	No. with AAA	Person-years	Multivariable HR*
**Hypertension**						
0	735	551 658	1.00 (reference)	371	155 252	1.00 (reference)
0.1–0.9 (0.3)	172	222 767	0.81 (0.68, 0.96)	78	55 416	0.77 (0.60, 0.99)
1.0–1.9 (1.0)	181	197 091	0.94 (0.80, 1.12)	87	52 281	0.85 (0.66, 1.08)
≥2.0 (2.0)	106	156 820	0.78 (0.63, 0.97)	51	35 769	0.77 (0.56, 1.05)
*P* for linear trend			0.042			0.019
Per cup			0.93 (0.88, 1.00)			0.88 (0.80, 0.98)
**Hypercholesterolaemia**						
0	826	615 760	1.00 (reference)	280	91 150	1.00 (reference)
0.1–0.9 (0.3)	193	244 261	0.81 (0.69, 0.95)	57	33 922	0.74 (0.55, 1.00)
1.0–1.9 (1.0)	199	219 037	0.89 (0.76, 1.05)	69	30 335	0.95 (0.72, 1.25)
≥2.0 (2.0)	121	172 882	0.77 (0.62, 0.94)	36	19 707	0.79 (0.54, 1.13)
*P* for non-linear trend						0.005
Per cup†						0.82 (0.67, 1.01)
*P* for linear trend			0.010			
Per cup			0.92 (0.86, 0.98)			

Values in parentheses are 95 per cent confidence intervals unless indicated otherwise. *Adjusted for age at study baseline (years, continuous), sex, education (primary, high school, or university), occupation (full-time, part-time, retired, disability pension, unemployed, or housewife), smoking status (never; ex-smoker: less than 20, 20–39, or at least 40 pack-years; or current: less than 20, 20–39, or at least 40 pack-years), BMI (less than 20.0, 20.0–24.9, 25.0–29.9, or at least 30.0 kg/m^2^), walking/cycling (less than 20, 20–40, 40–60, or over 60 min/day), history of diabetes (yes, no), and cardiovascular diseases (yes, no), family history of myocardial infarction (yes, no), aspirin use (yes, no), coffee consumption (no more than 1.0, 1.1–3.0, 3.1–5.0, or over 5.0 cups/day), sugar consumption (0, 0.1–1.0, 1.1–3.0, 3.1–5.0, or at least 5.1 teaspoons/day), modified Mediterranean diet score (points, continuous), and intake of energy (kcal/day, continuous). Missing data on educational level (0.5 per cent), smoking status (1.6 per cent), BMI (3.6 per cent), walking/cycling (8.7 per cent), and aspirin use (10.7 per cent) were included in the models as separate categories. †Calculated for linear range of association, that is tea consumption of at least one cup/day. AAA, abdominal aortic aneurysm; HR, hazard ratio.

## Discussion

In two population-based prospective cohorts of men and women, in a Swedish population with generally low tea consumption, tea drinking was inversely associated with risk of AAA. The association was more pronounced for ruptured than non-ruptured AAA, and in participants with hypertension and hypercholesterolaemia compared with those without these diseases. The inverse associations were observed in ex-smokers and never smokers, but not in current smokers.

The health benefits of tea consumption have been examined primarily in relation to CVD mortality and CVD events, and the findings of such studies are in line with those of the present analysis of AAA. In a meta-analysis^20^ of prospective studies it was reported that each cup of tea consumed per day (black and green) was associated with a 4 (95 per cent c.i. 2 to 6) per cent lower risk of CVD mortality and a 2 (0 to 4) per cent lower risk of CVD events.

The Swedish population is characterized by low tea consumption; over the years 1997–2019, tea consumption fluctuated between 0.3 and 0.4 kg per person per year. It was 0.3 kg per person in 1997 and 0.4 kg per person in 2009, whereas it dropped back to 0.3 kg per in 2019^31,32^.

Several potential mechanisms may be involved in the observed inverse association between tea consumption and AAA incidence. Tea, through its high content of flavonoids, may decrease systemic oxidative stress and inflammation which underlie AAA development^1^. It has been documented that flavonoids present in tea are effective radical scavengers that can reduce oxidative damage by scavenging oxygen free radicals^33^. Results of human intervention studies^33^ have demonstrated that green and black tea increase plasma antioxidant capacity, which may decrease oxidative damage of DNA and lipids. Another potential biological mechanism is the ability of tea flavonoids to reduce both systolic and diastolic BP^17,34^. Their essential role includes relaxing smooth muscle contraction, enhancing endothelial nitric oxide synthase activity and, as a result, improving endothelium-dependent vasorelaxation, reducing vascular inflammation, inhibiting renin activity, and reducing antivascular oxidative stress^35^. The role of tea in endothelial function seems to be crucial in reducing CVD risk, and green and black teas appear to have similar effects on improvement of flow-mediated dilatation^17^. One experimental study^36^ investigated the impact of specific green tea flavonoid (epigallocatechin-3-gallate) directly in relation to AAA progression. Rats supplemented with this green tea flavonoid had a lower level of AAA progression compared with controls; specifically, they had smaller abdominal aortic diameters, greater medial layer wall thickness, and higher elastin content^36^.

In this study, a statistically significant interaction between tea consumption and smoking status was observed in relation to AAA incidence (*P*_interaction_ < 0.001); an inverse association between tea consumption and AAA incidence was observed in ex-smokers and never smokers, but not in current smokers. It is possible that oxidative stress and systemic inflammation might be too high in current smokers to reduce AAA incidence by intake of tea flavonoids. A similar interaction was reported from a study^37^ on fruit consumption and risk of developing AAA; the HRs for ruptured AAA in the highest *versus* the lowest quartile of fruit consumption were 1.02 among never smokers, 0.42 among ex-smokers, and 0.39 among current smokers. Moreover, the inverse association between tea consumption and AAA risk was independent of the presence of hypertension. However, a stronger association was observed in participants diagnosed with than without hypertension (12 *versus* 7 per cent per cup of tea). Generally, tea is considered a beverage with the potential to reduce BP^35^, which may explain the stronger associations among participants with hypertension. Results of a meta-analysis^34^ of 25 RCTs indicated that long-term (at least 12 weeks) consumption of both green and black tea reduced systolic and diastolic BP. In addition, the observed inverse association seems to be independent of the presence of hypercholesterolaemia. The validity of registry data on hypercholesterolaemia is negatively affected by the fact that many patients with AAA are prescribed statins, even in the absence of high low-density lipoprotein (LDL) levels. However, it is not clear whether tea consumption is associated with lipid profile^8,9^. Generally, results of RCTs have suggested that black tea might not have beneficial effects on total cholesterol, high-density lipoprotein cholesterol, and LDL cholesterol^15,16^. In contrast, beneficial effect on lipid profile was observed in some experimental studies^38^; however, it should be emphasized that high doses of tea or tea components are usually employed in animal studies, which may affect the results.

Strengths of the present study include the large population-based cohorts, prospective design, use of validated FFQs to collect dietary information, high validity of self-reported tea consumption, as well as detailed information on smoking history and other potential confounders. Further strengths are the availability of data on long-term total tea consumption, and the complete follow-up of the study participants by linkage of cohorts with high-quality Swedish registers.

The study has limitations. The 1997 FFQ included only one question on total tea consumption, so it was not possible to conduct an analysis by specific types of tea in relation to risk of AAA in the baseline population or for long-term consumption. Although the self-reported frequency of total tea consumption has been shown to be highly valid^22^, some misclassification in self-reporting is inevitable. However, owing to the prospective design of the study, this type of misclassification would most likely attenuate true associations. Generally, making causal conclusions regarding risk factors for chronic diseases in observational studies is difficult because some potential confounders could be missed. It was noted that people who consumed at least two cups daily were better educated, had a lower BMI, were less likely to smoke, and less likely to have hypertension, diabetes, hypercholesterolaemia, or CVD; it is plausible that these features may reflect generally healthier behaviour or unobserved alternative social determinants (such as lower psychosocial stress), and may interact synergistically. In addition, healthier physical status might lead to less interaction with healthcare services and therefore a lower rate of detection of aneurysms in unscreened cohorts. Moreover, although the HRs were adjusted for many potential risk factors, a risk of potential residual confounding and its effect on attenuation of the observed associations cannot be excluded. Routine investigation of participants’ aortic diameter was limited to a subcohort of men, those who underwent AAA screening at the age of 65–75 years. Inference based on register-based cases could therefore have led to underdetection of asymptomatic AAA in participants classified as having non-diseased aortas. Such misclassification would most likely have led to underestimation of the risk estimates. Furthermore, as women in this study did not undergo population-based screening, it is possible that those with lower tea consumption were more prone to seeking medical attention, and thereby more likely to undergo opportunistic screening than women with high tea consumption. Such misclassification would most likely have led to overestimation of the risk reduction observed among women. However, the observed association with rupture as an outcome is unlikely to suffer from such bias.
